# Extreme Precipitation Events and Infectious Disease Risk: A Scoping Review and Framework for Infectious Respiratory Viruses

**DOI:** 10.3390/ijerph19010165

**Published:** 2021-12-24

**Authors:** Kyle T. Aune, Meghan F. Davis, Genee S. Smith

**Affiliations:** 1 Department of Environmental Health and Engineering, Johns Hopkins Bloomberg School of Public Health, Baltimore, MD 21205, USA; kaune1@jhu.edu; 2 Department of Molecular and Comparative Pathobiology, Johns Hopkins School of Medicine, Baltimore, MD 21205, USA; mdavis65@jhu.edu; 3 Division of Infectious Diseases, Johns Hopkins School of Medicine, Baltimore, MD 21205, USA

**Keywords:** climate change, rain, extreme weather, influenza, RSV, SARS-CoV-2, covid

## Abstract

Extreme precipitation events (EPE) change the natural and built environments and alter human behavior in ways that facilitate infectious disease transmission. EPEs are expected with high confidence to increase in frequency and are thus of great public health importance. This scoping review seeks to summarize the mechanisms and severity of impacts of EPEs on infectious diseases, to provide a conceptual framework for the influence of EPEs on infectious respiratory diseases, and to define areas of future study currently lacking in this field. The effects of EPEs are well-studied with respect to enteric, vector-borne, and allergic illness where they are shown to moderately increase risk of illness, but not well-understood in relation to infectious respiratory illness. We propose a framework for a similar influence of EPEs on infectious respiratory viruses through several plausible pathways: decreased UV radiation, increased ambient relative humidity, and changes to human behavior (increased time indoors and use of heating and cooling systems). However, limited work has evaluated meteorologic risk factors for infectious respiratory diseases. Future research is needed to evaluate the effects of EPEs on infectious respiratory diseases using individual-level case surveillance, fine spatial scales, and lag periods suited to the incubation periods of the disease under study, as well as a full characterization of susceptible, vulnerable, and sensitive population characteristics.

## 1. Introduction

During the past 40 years with the convening of the first Intergovernmental Panel on Climate Change in 1988 and through its five assessment reports, climatologists have reached consensus regarding the role of human activity in the increase of global temperatures and the acceleration of climate change [1,2,3,4,5]. Through increasing global temperatures, rising sea levels, challenges to food production, and population displacement, climate change significantly threatens public health. These threats come in the form of direct impacts of weather events, effects mediated through changing environmental systems, and effects mediated through changing human systems [6] and are projected by a World Health Organization risk assessment to cause 250,000 annual worldwide deaths between 2030 and 2050 mostly through malnutrition, infectious disease, and heat stress [7]. Many challenges and ecological changes are predicted by models of the future climate with varying levels of confidence, but among the highest-confidence predictions is the increase in the frequency of extreme precipitation events (EPEs) [8,9]. The most obvious effect of EPEs, flash flooding, cause significant direct harm to human health through injury and drowning, but these events are also associated with environmental and behavioral changes that foster transmission of many infectious diseases. These associations are well-established with regard to water- and vector-borne illness, but less well-studied in other classes of infectious diseases, particularly respiratory infections. To date, we find no published studies which summarize the impacts of EPEs on infectious disease transmission and public health. To fill this gap in the literature, this scoping review (1) summarizes the mechanisms and severity of impacts of EPEs on infectious diseases, (2) provides a conceptual framework for the influence of EPEs on infectious respiratory disease infection using influenza and SARS-CoV-2 as model pathogens, and (3) defines areas lacking in the current literature and recommends methodology for future investigations.

## 2. Extreme Precipitation Events (EPEs)

As the atmosphere has warmed over the past century, its water-carrying capacity has increased by about 7% per 1 degree Celsius of temperature increase [10]. In addition to further contributing to atmospheric greenhouse warming effects [11], increased water vapor has changed and is predicted to continue to impact global and regional precipitation, including patterns of heavy rainfall, drought, winter storms, tropical cyclones, and convective storm systems [9,12]. Since 1900, annual precipitation has increased globally [13]; throughout the United States [9,14], Eurasia, South America, and Australia [15]; and within specific North American regions [16,17,18]. Furthermore, the frequency and intensity of EPEs have increased as well [19].

EPEs are generally defined by setting a location-specific threshold and examining daily or multi-day precipitation totals for exceedance of that threshold; however, the thresholds can be calculated in many different ways that typically fall into two categories: theoretical and empirical. Theoretical distributions of precipitation are fitted based on the statistical theory of extreme values and can include adjustments for seasonal trends. Unfortunately, these EPE definitions require an a priori determination of an appropriate extreme value and are less directly comparable across regions [20]. On the other hand, empirical definitions of EPEs use the observed distribution of a location’s precipitation to determine the threshold, often the 90th, 95th, or 99th percentile to identify the top 10%, 5%, or 1% of precipitation events, respectively. Empirical thresholds are useful to detect temporal trends and are much more intuitively understood, making them appealing as a tool for effecting policy changes [20]. 

EPEs affect human life in many ways. While precipitation is not solely responsible for flooding, as other factors such as land use and engineering also contribute, intense precipitation is a primary cause of river and flash flooding [21]. Similarly, precipitation and storm surge from tropical cyclones and other weather extremes are largely responsible for many coastal flooding events [22,23]. The immediate effects of flooding are significant, with over 6,000 deaths occurring in the United States alone from 1959 to 2005 [24,25] and over 500,000 deaths, 360,000 injuries, and 2.8 billion displaced individuals worldwide have been attributed to flooding from 1980 to 2009 [26], and 1.65 billion individuals affected and over 100,000 killed worldwide from 2000–2019 [27], though these are likely underestimates of the true burden. Furthermore, beyond the immediate life-threatening effects of flash flooding and storm surge, long-term psychological harm can often develop through the trauma of experiencing natural disasters [28] and persistent mold growth and intrusion continue to trouble individuals affected by flooding [29].

Extreme precipitation leading to flooding also causes damage to many aspects of the built environment including private property, public infrastructure, and agriculture. In the United States, annual damages to property and crops from flooding averaged about $6 billion per year over the latter half of the 20th century [30], and the National Weather Service reported direct damages from flooding of nearly $80 billion from 2015–2019 [31]. Worldwide, economic losses due to flooding are estimated at $651 billion from 2000–2019 [27]. Extreme precipitation, regardless of whether flooding results, can also overwhelm municipal water systems. While many municipalities have separate systems for handling drinking, waste, and storm water, combined sewer overflows are a significant concern during EPEs for the nearly 860 communities in the United States alone that rely on these outdated systems [32]. Not only are there direct costs associated with these combined sewer overflows, but due to the increasing threat of more frequent EPEs, municipalities are also faced with the cost of upgrading to more resilient systems to handle storm water separately from contaminated wastewater. 

Furthermore, extreme precipitation can alter the natural environment in more subtle ways during extreme events and in subsequent days and weeks. Changing patterns of EPEs alter the availability of moisture in soil, leading to some areas becoming more arid while others experience higher soil moisture [33]. At the same time, relative humidity rises during precipitation events and continues to rise following precipitation due to evaporation. 

In response to these effects on the natural and built environments, EPEs also modify human behavior. While it is intuitive to understand that EPEs influence the likelihood that people will stay indoors in their homes rather than spend time outdoors or in public, generalizable findings in the scientific literature describing these effects remains sparse. A study of German schoolchildren found that the number of unique contacts children made was lower during rainy days [34], and a study of Belgian schoolchildren confirmed these findings but also found that the time spent with each contact was longer during precipitation events [35]. Information regarding contact networks during precipitation in the context of infection transmission dynamics is limited in the public health literature to these investigations of self-report in European school children; however, the potential for EPEs to influence behavior is well-studied by retail economists. As early as 1951, economists have identified a relationship between precipitation and lower retail sales volume which has been attributed to the fact that it is uncomfortable or impossible for adults and families to leave home for a retail shopping trip during certain weather conditions, such as heavy rain or snow or temperature extremes [36]. In addition to discomfort outside the home, the alterations to the natural environmental brought about during EPEs, namely higher humidity and lower temperature, lead to a higher demand of the warming and de-humidifying effects of indoor heating, ventilation, and air conditioning (HVAC) systems. When weather events drive populations indoors, the indoor built environment, therefore, can play a significant role in fostering or hindering transmission of infectious diseases, especially through the use of effective, efficient HVAC systems. However, availability and use of HVAC units, as well as their efficiency and effectiveness in removing infectious microbes, are differential with respect to household income and their effect on indoor environmental conditions during precipitation events may vary among strata of socioeconomic status.

## 3. Effects of EPEs on Infection Dynamics

### 3.1. Enteric Illnesses

Due to their impact on the natural and built environments and on human behavior, EPEs provide a number of mechanisms that could be expected to enhance transmission of infectious enteric illness. Heavy precipitation can overwhelm combined municipal sewer outflow systems backing sewage into homes and businesses, while dispersed surface water from flooding can transport bacterial and viral contaminants from wastewater treatment and animal agriculture facilities to human communities. Furthermore, severe flooding resulting from EPEs often leads to temporary population evacuation and displacement that can result in crowding and overwhelming of the local wastewater handling infrastructure in the area receiving the displaced populations. These pathways for transmission have led to documented increases in incidence of cholera [37,38,39], shigellosis [38], typhoid [40], enterovirus [41], cryptosporidiosis [42], campylobacterosis [43], and salmonellosis [44] following EPEs or heavy sustained rainfall events. This phenomenon is not geographically, climatically, or developmentally isolated to any one specific region of the world–enteric illness outbreaks following heavy precipitation have been observed on all continents other than Antarctica; in tropical, sub-tropical, and temperate climates; and in low-, middle-, and high-income countries, including the United States. These associations range from moderate increases in monthly incidence rates when examined at county- and country-level scales [38,39,40,42,43,44] to strong increases in the odds of infection when examined at the individual level [37,41]. Significant associations are seen between these enteric illnesses and total precipitation amounts as well as extreme events when annual and monthly rates are examined [38,39,42,43,44], as well as when individual risk is considered with appropriate temporal lag periods [37,40,41]. These lag periods vary by pathogen according to their respective incubation periods and range from several days to several weeks. The wide variation in effective lag periods and measures of association point to the strength of study methods correlating daily precipitation measurements with individual disease cases using pathogen-specific lag periods.

### 3.2. Vector-Borne Illnesses

Natural and built environment changes and altered human behavior from EPEs also favor increased transmission of vector-borne infectious diseases, particularly with regard to mosquito vectors [45,46,47,48,49,50,51,52,53,54], though tick population numbers have also been linked with wetter environmental conditions [55]. While it was originally believed that heavy rainfall and flooding would flush out mosquito larval habitats leading to overall decreases in mosquito populations and associated illness following EPEs, the opposite has been repeatedly observed. While flowing water during EPEs will wash away mosquito larvae and eggs, receding water after EPEs leaves behind new habitat for mosquitoes that quickly return and increase in numbers [56]. Due to the abundance of suitable breeding sites following heavy precipitation and flooding events, rates of mosquito-borne illness increase significantly, even in areas that typically experienced only low risk of disease at a typical lag of around 8 weeks after precipitation events [57]. In addition to the effects brought about by increased breeding of mosquito vectors, behavioral changes during and immediately after heavy rainfall events can also contribute to more immediate increases in disease risk. Increased indoor behavior during EPEs and displacement and crowding associated with severe EPEs and flooding events pose a significant risk of vector-borne illness transmission. This risk is especially important considering diseases that are spread by endophilic, diurnal mosquitoes that take blood meals more often indoors during the day, such as mosquitoes that belong to the *Aedes* genus responsible for spread of the flavivirus causing Dengue fever. In fact, EPEs have been associated with increases in incidence of Dengue fever [46,47,48,49], malaria [50,51], West Nile Virus [52], and Japanese encephalovirus [53,54]. Once again, these associations between heavy precipitation events and vector-borne illness are widespread and have been described in Asia, South America, North America, and Europe [46,47,48,49,50,51,52,53,54]. These associations range from moderate increases in monthly and weekly incidence rates [46,47,48,49,50,51,53,54] to strong increases in the odds of infection when examined at the individual level [52]. The strongest effects are seen at lag periods of around one to two months [46,48,50,53,54], but associations have been identified at lag periods that range from two weeks to three months [47,51,52]. Contrasted with the varying effective lag times of enteric disease dynamics, the mechanism for EPEs’ effects on vector-borne diseases is the same regardless of pathogen. Therefore, differences in measures of association across lag periods appear less due to differences in the pathogen characteristics, but rather due to differences in study methodology.

### 3.3. Allergic Illnesses

In addition to the widely demonstrated effects of EPEs on enteric and vector-borne illnesses, research also suggests that EPEs may alter the natural environment in ways that facilitate acute and chronic allergic respiratory illness. Standing surface water and soil saturation during EPEs have been observed to result in increased fungal spore production and release [58]. In addition, it is believed that rainfall causes allergenic pollen granules to osmotically rupture into respirable bioaerosols [59]. Both of these environmental alterations by EPEs have led to observation of epidemics of “thunderstorm asthma”, as well as increased rates of allergic rhinitis following EPEs and flooding events [60,61,62,63,64,65]. Many of these associations are described as clinical case series [60,61], but a moderate statistical measure of association is described when daily hospital case counts are compared with meteorologic factors [64], and very strong measures of association are described when these effects are examined at the individual level [62,63]. These effects are generally more acute in nature, with lags ranging from 0–7 days, but chronic allergic respiratory illness is also common in indoor areas experiencing frequent flooding and mold growth [66]. Though not infectious, these phenomena lend credence to the idea that EPEs and heavy precipitation can cause negative respiratory health outcomes.

## 4. A Framework to Evaluate How EPEs Influence Infectious Respiratory Disease

**Figure 1:**
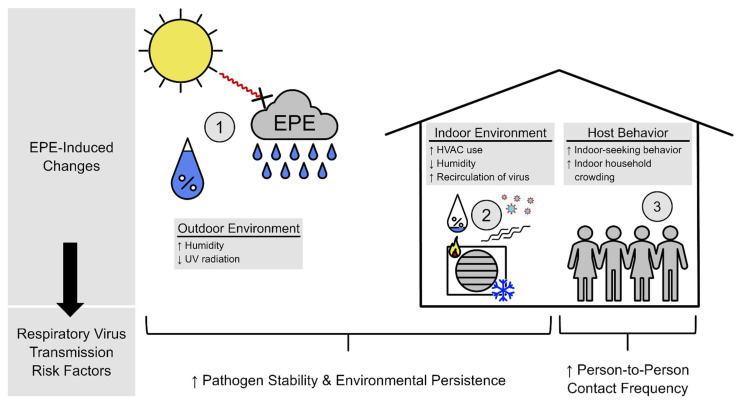
Conceptual framework of the impact of extreme precipitation events (EPEs) on infectious respiratory viruses. EPEs alter (1) the natural environment by increasing humidity and decreasing UV radiation, (2) indoor environments by increasing heating, ventilation, and air conditioning (HVAC) system use which in turn lowers humidity and increases recirculation of virus-laden droplets and aerosols, and (3) human host behavior by prompting increased indoor-seeking behavior and household crowding. Changes to the outdoor and indoor environments increase pathogen stability and persistence in outdoor and indoor environments and changes in host behavior increase person-to-person contact frequency, two important factors in favoring increased transmission dynamics.

In comparison to enteric, vector-borne, and allergic illnesses, infectious respiratory diseases share some of the same transmission dynamic mechanisms, yet there is a dearth of research that examines this process. In Figure 1, we provide a conceptual framework outlining potential pathways by which EPEs contribute to increases in infectious respiratory disease transmission through changes in the natural environment, built environment, and human behavior. We propose EPEs induce environmental and behavioral changes favoring respiratory viral transmission in the following ways:

1. Outdoor environmental risk factors: Cloud coverage during EPEs decreases environmental deactivation of microbes, particularly viruses, via ultraviolet (UV) irradiation
2. Indoor environmental risk factors: HVAC use during EPEs decreases indoor humidity
3. Host behavioral risk factors: Individuals exhibit indoor-seeking behavior during EPEs

### 4.1. Outdoor and Indoor Environmental Risk Factors

Transmission of infectious respiratory viruses depends on contact between a susceptible individual and a viable virion shed by an infectious individual, and the likelihood of this contact event occurring and resulting in a new infection is dependent upon a number of environmental factors. Common routes of exposure for respiratory viruses are inhalation of infectious respiratory aerosols and droplets, and for a respiratory virus to be inhaled, the droplets containing it must remain suspended in the air in an individual’s breathing zone (i.e., at a height near a susceptible individual’s nose and mouth) [67]. The more time a virion spends in the breathing zone, the greater chance it has of exposing the host to an infectious dose. Respiratory droplets large enough to be sufficiently affected by gravity (>100 µm in diameter) settle from the respirable zone to surfaces quickly where they are unlikely to cause infection via inhalation (unless resuspended in a viable state from a non-porous surface). Infectious aerosols are generally defined as droplets smaller than 10 µm in diameter and are able to remain suspended for minutes to hours, while inspirable droplets range in size from 10–100 µm. Aerosols and inspirable droplets remain suspended in the air and slowly evaporate until completely desiccated and any virions contained within are inactivated [68]. Therefore, the respirability of infectious droplets depends on a balance between droplet size and evaporation rate, the former not being affected by environmental factors, but the latter being very much affected by vapor pressure, a simple mathematical relationship between temperature and relative humidity in the surrounding environment [69]. Transmission probabilities are highest in low vapor pressure environments [70], which are conditions created by cold temperature, low relative humidity, or a combination thereof [71,72]. While EPEs result in lower relative humidity in ambient environments, HVAC systems remove humidity from indoor air, lowering indoor vapor pressure and thus slowing droplet evaporation and increasing the time infectious droplets remain suspended and respirable in indoor air [73]. Furthermore, droplets and aerosols able to avoid evaporation in indoor air are recirculated by HVAC systems that often do not have air filters capable of removing such small particles [74], effectively concentrating the air with infectious virions. Another important environmental factor that influences airborne viral survival is exposure to UV radiation, typically sunlight. Solar as well as artificial UV irradiation has been shown to inactivate many microbes including viruses such as influenza, RSV, and the coronavirus responsible for COVID-19 [68,73,75,76,77]. EPEs coincide with significant cloud coverage which decreases insolation and UV irradiance in both outdoor and indoor environments, lengthening the environmental survival time of infectious viruses. While transmission dynamics are complicated processes, the effects that EPEs have on the natural and built environment enhance these processes and provide an opportunity for increased transmission.

### 4.2. Behavioral Risk Factors

In addition to respiratory droplets, the remaining mechanisms of infectious respiratory virus transmission are direct contact, indirect (fomite) contact, and inhalation of airborne droplet nuclei (<5 µm in diameter) [67]. The likelihood of a new infection occurring in a susceptible individual is governed by the rate at which that individual comes into contact with infectious virions [78]. Assuming constant environmental conditions, the primary mechanism of increasing the frequency of these encounters is increasing the magnitude of infectious virions in the vicinity. With the exception of airborne transmission, which varies among respiratory viruses in its importance among transmission pathways, direct contact, indirect contact, and respiratory droplet transmission are enhanced through close contact between infectious and susceptible individuals in conditions that slow or prevent airborne dilution or surface inactivation via UV radiation, conditions best accomplished through indoor crowding. Indoor crowding was noticed by the ancient Greeks as a risk factor for illness, was implicated as a cause of the 1918 influenza pandemic in military barracks and hospitals during World War I [79], and indeed close indoor contact is a leading risk factor for transmission of all respiratory viruses including SARS-CoV-2, influenza, measles, respiratory syncytial virus (RSV), and others [73,80,81,82,83,84,85,86,87]. National and local ordinances enforcing physical distancing in indoor settings during the SARS-CoV-2 pandemic have been effective at decreasing transmission rates, pointing to the importance of indoor crowding in facilitation of viral transmission [88,89].

### 4.3. Support in Seasonal Patterns of Infectious Respiratory Disease

Many respiratory viruses exhibit annual seasonal epidemics, including measles, RSV, coronaviruses responsible for the common cold, and most notably influenza [71,80,82,84,90,91]. Three factors influence these regular seasonal peaks and dips: pathogen circulation, environmental characteristics, and host behavior [92]. Viral migration or, in the case of influenza and other pathogens that undergo rapid mutation, antigenic drift, may explain some of the variance in infections over time, but they do not sufficiently account for the regularity in the timing of seasonal peaks in infection. Seasonal variations in the environmental and behavioral risk factors described above therefore must account for a significant amount of regularity in infection patterns. Indeed, annual trends in temperature and humidity have been associated with rates of influenza and RSV [93,94,95,96], and these trends are regionally specific. In temperate zones, regular seasonality is exhibited in many respiratory viruses with strong peaks in the cold, low-humidity winter months, and these trends are similar for temperate zones in the northern or southern hemisphere [84,97]. While influenza and other respiratory viruses occur in epidemics in subtropical and tropical zones, the seasonal patterns are not uniformly as strong and regular as in temperate zones [82,86]. Some tropical areas exhibit seasonality during warm months, others exhibit seasonality during cooler months, and some do not exhibit regular seasonal epidemics [67]. This relative disagreement in the strength of environmental factors alone in their ability to account for seasonal variation leaves room for the contribution of seasonal differences in host behavior to affect epidemic timing. A unifying feature of the annual periods of increased respiratory viral transmission regardless of climate zone is the presence of environmental factors that drive indoor behavior and crowding. In temperate zones, these factors are primarily driven by cold temperatures. In tropical climates, areas experiencing seasonal epidemics usually have peaks in respiratory infections during the rainy season [95]. While there is no consensus as to whether either environmental or behavioral factors are the primary driving force behind the regularity of seasonal respiratory infections, it is clear that their interaction is a significant component.

### 4.4. Existing Evidence and Remaining Unknowns

A brief review of literature describing the relationships between precipitation measures and respiratory virus infections is presented in Table 1. These studies were identified through searching for common respiratory virus keywords and including some form of precipitation (i.e., “precipitation”, “rain”, “snow”) and no exclusions were made with regard to temporal or geographic setting. Studies in Table 1 are organized by (1) pathogen, (2) precipitation measure, then (3) finding. The association between precipitation and influenza transmission has been studied worldwide; however, a substantial focus has been placed on this relationship in tropical climates. Additionally, nearly all of the studies identified used some level of aggregation in their methodology–either spatially aggregating influenza cases to a city or county incidence rate or temporally aggregating precipitation to a weekly or monthly sum. The studies that aggregated rainfall to the weekly or monthly level relied on cross-sectional or serial cross-sectional surveillance to arrive at incidence rates, and, due to temporal aggregation of case counts and precipitation measurements, most cannot consider lag periods between precipitation on the date of infection and the date of a positive influenza test. Two notable exceptions to this shortcoming are the findings of case-crossover studies by Murray et al. [98], who examined the total precipitation in the week preceding a positive test, and Smith et al. [99], who examined the relationship between EPEs with a lag of six days prior to an emergency room visit [99]. These studies conducted at the individual level identify some of the strongest measures of association among the identified studies [98,99]. 

In the reviewed literature, measures of association between precipitation and influenza infection were mixed but generally positive among the eight reported studies [95,98,99,100,101,102,103,104]. The inverse relationship described by Nisar et al. and Stark et al. represent investigations of measures of precipitation aggregated to monthly totals which, while meaningful for describing typical spatiotemporal risk or seasonality of influenza due to precipitation, are limited by a long time scale that washes out the heterogeneity of day-to-day changes in risk of influenza due to precipitation–daily variations in precipitation would regress to a mean value and days without precipitation at all would drag average daily values toward zero [102,104]. Three studies relied on unadjusted correlation coefficients to make conclusions about the relationship between influenza and precipitation without accounting or controlling for the contribution of other confounding factors [95,100,103] and every study considering precipitation as a continuous variable has modeled it linearly. While it is possible that there is a range of precipitation amounts over which a linear relationship between total precipitation and influenza transmission might be plausible, it is more likely that the association between precipitation and influenza transmission is non-linear and might be more accurately described by a threshold. A basic assumption of linear regression modeling is for the existence of a linear relationship between the dependent and independent variables—an assumption that may be violated in these cases as we propose the effect on infection dynamics of a change in daily precipitation from, for example, 0 cm to 1 cm is likely to be stronger than a change from 10 cm to 11 cm. 

It is important to note that while Table 1 describes the effect of various measures of precipitation on the risk of respiratory virus infection, only one study has considered the potential that threshold-exceeding events such as EPEs might influence respiratory virus infection. This investigation found a significant association between EPEs and emergency room visits for influenza over a seven-year period in Massachusetts, USA [99]. While an important finding, these results are limited in their generalizability because the population presenting to emergency departments is likely either more severely ill and requiring emergent care from a progressed form of illness or, due to low income or unemployment, uninsured or otherwise without access to a primary care physician for care related to a moderate case of illness. Therefore, emergency room visits may not serve as a good surrogate of the true influenza burden in a population. Furthermore, a significant amount of precipitation during influenza season falls as snow in Massachusetts, and unfortunately, this study was unable to characterize the type of precipitation that occurred in extreme events. However, the highest frequency of EPEs occurred during the winter when 30–50% of winter precipitation in the study area falls as snow or mixed snow, ice, or rain [105] thereby limiting the geographic generalizability of these findings. However, while this area may experience more snow and therefore be relatively colder than some other areas, the authors do include and control for daily temperature and season in their conditional logistic models, likely capturing and accounting for the potential confounding actions of cold temperatures that would coincidentally drive indoor-seeking behavior during winter extreme precipitation events.

In addition to influenza, a single investigation of meteorological risk factors of non-SARS, non-MERS coronavirus infections in Germany described a significantly positive association between daily precipitation and positive coronavirus tests after a lag period of 10 days [106]. This is in line with findings of influenza investigations supportive of a lagged effect of precipitation on infection risk tied to the natural history of the infection, but points to the dearth of high quality, generalizable studies in the current literature.

Of particular public health interest is any association between meteorological factors and risk of SARS-CoV-2 infection that could aid in caseload prediction for public health agencies and healthcare providers. While temperature and humidity are often identified in these investigations as significantly associated with SARS-CoV-2 infection, there is not consensus among the published literature on the role precipitation might play. While many studies find no significant association between precipitation and SARS-CoV-2 risk [107,108,109,110], Sarkodie et al. demonstrate moderate positive associations [111], and daily total precipitation is described as a significantly protective effect by Chien et al. and Menobo et al. [112,113]. The null findings reported by many are likely affected by the short observation period and a lack of variation in precipitation measures; longer observation periods might elucidate significant associations. Furthermore, only the investigation by Chien et al. [112] incorporated a lag period. However, the only lag period considered was 3 days—slightly shorter than the established median incubation period for COVID-19 of 4–5 days [114,115,116]. Two studies averaged meteorological factors in the two weeks preceding the date of interest with a goal of describing the “typical” meteorology during transmission events leading to a daily incident case count [108,110], but this approach is inappropriate to determine exposure to typical precipitation when precipitation is measured as an absolute amount (i.e., mm or cm), as carried out in these studies, since days with no precipitation will zero-weight the mean, systematically underestimating exposure to precipitation. The remaining studies correlated infection rates with same-day meteorologic conditions—an approach unlikely to describe meteorologic factors associated with transmission given the lag between the transmission event and onset of symptoms. Finally, these studies are all limited by the types of exposure and outcome data used. With the exception of the investigation by Ward et al., each study correlates daily case counts with meteorologic values measured at single or very few observation stations. These studies assess meteorology at a spatial scale ranging from city, to county, to state, to country and, as such, suffer from varying degrees of exposure misclassification as there is significant variation in daily meteorology among these levels of geography.

While the inverse associations between precipitation levels and COVID-19 cases are at odds with the proposed mechanisms whereby precipitation might influence infectious respiratory virus transmission described by Figure 1, it is necessary to note important differences in behavioral factors in effect during the COVID-19 pandemic that might explain these results. Stay-at-home orders, mandatory business closures, remote learning for children and adolescents, and a dramatic increase in the number of adults working from home have all been implemented with the goal of reducing person-to-person contacts that could result in infection. This has resulted in much more indoor-seeking behavior, but these contact isolation practices generally prevent social mixing and limit person-to-person contacts to within households. Therefore, while the immediate effect of encouraging indoor seeking behavior would be the same during an EPE, the effect of precipitation on populations under COVID-19 restrictions might be substantially different compared to the pre-pandemic period. Since pre-pandemic populations exhibited much more social mixing with higher numbers of person-to-person contacts on a typical day, indoor seeking behavior during a precipitation event would bring household contacts, some of whom may have had infectious contacts in the preceding days, in close indoor proximity to one another where secondary household infections could occur. Contrastingly, since indoor seeking behaviors within households are already high due to COVID-19 restrictions, a precipitation event is likely sufficient to encourage indoor-seeking behaviors among the remaining population reluctant to follow physical distancing guidelines or whose occupations ordinarily require work outside the home, preventing social mixing and primary infections. As evidence, seasonal influenza cases were 99% lower than historic rates during the 2020–2021 season worldwide and the influenza B/Yamagata lineage may have disappeared due to these behavioral changes [117,118].

## 5. Future Directions

When considering the potential health implications of more frequent EPEs related to climate change, it is imperative that susceptible, vulnerable, and sensitive populations are addressed – susceptible populations being those with biologic factors that make them more at risk of infection or serious illness, vulnerable populations being those at higher risk due to social or environmental factors, and sensitive populations being those that are both susceptible and vulnerable. There are a number of biological risk factors for infectious respiratory viruses related to age, immune status, and comorbidities. These include older age, pregnancy, asthma and other reactive airway disorders, neurologic and neurodevelopmental conditions, blood disorders and hemoglobinopathies, chronic lung disease, endocrine disorders, heart disease, kidney disorders, liver disorders, metabolic disorders, BMI > 40, cancer, and immunocompromising conditions or immune-weakening medication use. In addition, smokers, people living in nursing homes or long-term care facilities, and, in the United States, certain racial and ethnic groups including non-Hispanic black people, Hispanic or Latino people, and American Indian and Alaska Native people are more vulnerable to initial infection or more severe infection [119,120,121]. These factors can operate independently or together to make individuals more susceptible or vulnerable to initial infection by lowering the infectious dose or dampening the innate or adaptive immune response, some factors place infected individuals at increased risk of more severe disease, and many exist along modifiable psychosocial stress pathways [122]. As many of these factors represent populations who would experience the strongest health effects of EPEs, identification of susceptible, vulnerable, and sensitive populations provides the public health community a significant opportunity to develop specific mitigation strategies with maximum impact potential in preventing morbidity and mortality—a need identified in a report by the U.S. Interagency Working Group on Climate Change and Health [123].

Future research into the effects EPEs have on influenza, other infectious respiratory illness including COVID-19, and other non-respiratory illness is important given the effects of climate change on the frequency and intensity of precipitation. Furthermore, it is necessary that threshold-based definitions of precipitation are examined in future research. Not only do these measurements more accurately capture exposure to precipitation sufficient to alter transmission dynamics in a way that absolute precipitation treated continuously is unable to do, but these methods allow researchers to address the conditions predicted by future climate models whereby total annual precipitation does not increase by a significant amount, but instead precipitation is concentrated in a more frequent extreme events [9]. However, a variety of thresholds should be explored to determine whether exceeding locally relative thresholds (e.g., 99th percentile) or a range of absolute thresholds (e.g., 2.54 cm) is most meaningful for affecting respiratory viral transmission. In addition, many existing studies are prone to exposure misclassification due to spatial or temporal aggregation of case surveillance and meteorologic measurements. Exposure assessment is most accurate when performed on fine spatial and temporal scales that allow for the capture of variability in weather patterns over relatively small areas and for the exploration of pathogen-specific temporal lags between exposure and the measured outcome. Exposure misclassification is likely non-differential and therefore would bias measures of association toward the null, evidenced by the stronger results in existing studies using individual level data and daily meteorologic measurements.

The conceptual framework presented in the present review provides a number of mechanisms whereby EPEs might influence infectious respiratory illnesses, but many exist along multi-step causal pathways where EPEs influence a mediator (such as indoor seeking behavior), which impacts transmission probability. Therefore, in addition to examining the overall effect of EPEs on infectious respiratory illness, future studies should also seek to quantify the strength of these proposed mediating pathways by characterizing changes of indoor-seeking behavior, indoor crowding, heating and cooling system usage, HVAC filtration abilities, and indoor temperature and humidity during EPEs. Much of the literature describing the impact of precipitation on respiratory infections is focused in tropical areas that experience seasonal rain patterns. However, subtropical and temperate patterns of illness currently influenced by cold weather may be complicated by precipitation in the near future and could eventually be primarily driven by seasonal precipitation by the end of the 21st century as temperature and precipitation begin to demonstrate more tropical weather patterns. Studies that address these needs will provide tremendous public health benefits as seasonal patterns of illness in temperate zones will likely undergo changes as part of the “expanding of the tropics” predicted by climate models [124]. While pandemic respiratory viruses deserve unique strategies to mitigate transmission and minimize severe illness and death, a fuller understanding of the contribution of precipitation and EPEs on seasonal and sporadic risk will mitigate the impacts of interpandemic respiratory illness by informing the timing and intensity of prevention and control measures and informing the timing of surveillance, production, and administration of vaccine prevention programs and, with tailored mitigation strategies for susceptible, vulnerable, and sensitive populations, can attempt to prevent or lessen some of the earliest human health effects of climate change.

## 6. Conclusions

Precipitation and EPEs have increased over time and will continue to increase due to climate change and increasing global temperatures. Much of this precipitation is expected to occur in the form of more intense and frequent extreme events. The effects of precipitation and EPEs alter the natural environment, built environment, and human behaviors in ways that facilitate enhanced transmission of infectious diseases and there is abundant evidence of these effects on the burden of enteric illness, vector-borne illness, and allergic illness. There is also moderate evidence of these effects on enhancing infectious respiratory virus transmission that is supported by a conceptual framework. However, published associations between EPEs and infectious respiratory virus transmission are likely weakened by inadequate study methodology. Future studies into these effects should consider threshold-exceedance-based definitions of precipitation exposure to (1) more accurately describe the mechanisms of EPEs’ effects on transmission dynamics and (2) to more accurately capture the form of precipitation events predicted to occur in the coming century due to climate change.

## Figures and Tables

**Table 1:** Review of published literature investigating the association between precipitation and respiratory virus infection. All measures of association represent an association between incident cases of the pathogen with the specific precipitation measure under study. 95% confidence intervals are provided in parentheses after ratio estimates. Studies are organized by (1) pathogen, (2) precipitation measure, then (3) finding.

Study	Pathogen	Setting	Time Period	Precipitation Measure	Finding
Smith et al., 2017	Influenza	Massachusetts, USA	7 years (2002–2008)	Daily extreme events (top 1%) by city	Positive association OR = 1.20 (1.14–1.26)
Chew et al., 1998	Influenza	Singapore, Singapore	5 years (1990–1994)	Daily city total (mm)	Positive association ρ = 0.08 (*p* < 0.05)
Murray et al., 2012	Influenza	Kamalapur, Bangladesh	92 days (2005)	Weekly city total (inch)	Positive association OR = 2.97 (1.87–4.70)
Gomez-Barroso et al., 2017	Influenza	Spain	6 years (2010–2015)	Weekly total by city (per 50 mm)	Positive association RR = 1.37 (1.20–1.56)
Agrawal et al., 2009	Influenza	Kolkata, India	2 years (2007–2008)	Monthly city total (mm)	Positive association r = 0.90 (*p* < 0.001)
Rao and Banerjee, 1993	Influenza	Pune, India	13 years (1978–1990)	Monthly city total (mm)	Positive association r = 0.70 (*p* < 0.05)
Nisar et al., 2019	Influenza	Islamabad and Multan, Pakistan	5 years (2012–2016)	Monthly total by city (mm)	Negative association r = −0.3 (*p* = 0.02)
Stark et al., 2012	Influenza	Pennsylvania, USA	7 years (2003–2009)	Mean monthly total by county (inch)	Negative association OR = 0.52 (0.28–0.94)
Anastasiou et al., 2021	Non-SARS, non-MERS coronaviruses	Essen, Germany	7 years (2013–2019)	Daily city total (per 5 mm)	Positive association OR = 1.21 (1.07–1.36)
Sarkodie et al., 2020	SARS-CoV-2	20 countries worldwide	97 days (2020)	Daily mean total by country (mm)	Positive association β = 1.01 (*p* < 0.001)
Bashir et al., 2020	SARS-CoV-2	New York City, USA	43 days (2020)	Daily city total (mm)	No association τ = −0.22 (*p* > 0.1)
To et al., 2020	SARS-CoV-2	4 Canadian provinces	114 days (2020)	Daily total by region (mm)	No association β = −27.1 (*p* = 0.38)
Tosepu et al., 2020	SARS-CoV-2	Jakarta, Indonesia	89 days (2020)	Daily city total (mm)	No association ρ = 0.14 (*p* > 0.05)
Ward et al., 2020	SARS-CoV-2	New South Wales, Australia	49 days (2020)	Median daily total by postal code (mm)	No association r = −0.03 (*p* = 0.66)
Chien et al., 2020	SARS-CoV-2	50 counties in the USA	37 days (2020)	Daily total by county (inch)	Negative association RR = 0.93 (0.92–0.94)
Menobo, 2020	SARS-CoV-2	Oslo, Norway	64 days (2020)	Daily city total (mm)	Negative association r = −0.285 (*p* = 0.022)

r = Pearson correlation coefficient; ρ = Spearman rank sum coefficient; RR = risk ratio; OR = odds radio; τ = Kendall rank coefficient; β = linear regression coefficient.
